# Early State Research on Antifungal Natural Products 

**DOI:** 10.3390/molecules19032925

**Published:** 2014-03-07

**Authors:** Melyssa Negri, Tânia P. Salci, Cristiane S. Shinobu-Mesquita, Isis R. G. Capoci, Terezinha I. E. Svidzinski, Erika Seki Kioshima

**Affiliations:** Universidade Estadual de Maringá, Micologia Médica, Av. Colombo, 5790, CEP 87020-900, Brazil; E-Mails: melyssanegri@gmail.com (M.N.); taniasalci@gmail.com (T.P.S.); cristianeshinobu@gmail.com (C.S.S.-M.); isiscapoci@hotmail.com (I.R.G.C.); tiesvidzinski@uem.br (T.I.E.S.)

**Keywords:** antifungal, natural products, essential oils, extracts, fungi

## Abstract

Nosocomial infections caused by fungi have increased greatly in recent years, mainly due to the rising number of immunocompromised patients. However, the available antifungal therapeutic arsenal is limited, and the development of new drugs has been slow. Therefore, the search for alternative drugs with low resistance rates and fewer side effects remains a major challenge. Plants produce a variety of medicinal components that can inhibit pathogen growth. Studies of plant species have been conducted to evaluate the characteristics of natural drug products, including their sustainability, affordability, and antimicrobial activity. A considerable number of studies of medicinal plants and alternative compounds, such as secondary metabolites, phenolic compounds, essential oils and extracts, have been performed. Thus, this review discusses the history of the antifungal arsenal, surveys natural products with potential antifungal activity, discusses strategies to develop derivatives of natural products, and presents perspectives on the development of novel antifungal drug candidates.

## 1. Introduction

Fungal participation in the aetiology of infections has increased considerably [1,2]. However, as medical technology has improved, the survival of patients with severe and life-threatening illnesses has led to a rapid increase in the immunosuppressed population [3]. These changes are correlated with a substantial increase in the rate of invasive fungal infections (IFIs). Moreover, drug-resistant strains are emerging, and the number of infections by intrinsically drug-resistant species has increased rapidly [4]. Despite the constant introduction of new and effective synthetic drugs to the market, medicinal plants, which are the historical basis of therapeutic health care, represent an alternative that is economical, accessible, and applicable to various pathologies, particularly in developing countries [5].

Therefore, parallel to the development of synthetic drugs, substantial attention has focused on natural products with antifungal properties, which has stimulated the search for therapeutic alternatives [6,7]. Therapies commonly called alternative, complementary and homemade have been used for centuries, and studies have intensively investigated plant species with medicinal properties to assess the feasibility, sustainability and affordability of the use of natural drugs [8,9].

Plants produce a variety of medicinal components that can inhibit the growth of pathogens, and a considerable number of studies have been conducted to evaluate the antimicrobial activity of extracts and essential oils of medicinal plants [10,11]. Natural products have diverse chemical characteristics that can influence the evaluation of antifungal activity, such as the release of active constituents, solubility, stability, absorption and dissolution. Thus, the focus of this review was to briefly discuss the history of the antifungal arsenal, survey natural products with potential antifungal activity, discuss strategies to develop natural product derivatives, and present perspectives on the development of novel antifungal and novel drug targets.

## 2. A Brief History of Antifungal Agents

In the first decades of the 20th century, there was great concern regarding dermatophytoses and thrush. Consequently, therapeutic attempts were directed toward the treatment of these infections. In 1935, Wieder [12] recognised that the number of fungal dermatoses was growing, necessitating treatment with drugs specific for fungal dermatitis. Since that time, the interest in antifungal clinical therapy has gradually increased. Therapies have been limited to non-specific remedies such as iodide; mercury; benzoic and salicylic acids; phenol derivatives; undecylenic acid; methyl violet; sulphonamide derivatives; and other noxious agents, including bromine, potassium permanganate and oil of turpentine with olive oil [13,14,15].

Griseofulvin, a compound derived from *Penicillium griseofulvum,* was the earliest chemical claimed to show selective inhibitory activity against fungi and was widely used to treat superficial fungal infections [16,17]. Nystatin, initially called fungicin, was the first polyenic compound with known antifungal activity [18]. Other studies were performed by the same researchers to prove its antifungal efficacy and utility [19,20].

In 1955, an important advance was amphotericin B, which is considered the most effective antifungal drug, even with the adverse side effects such as severe dose-dependent toxicity, including renal impairment and hypokalaemia [21]. Currently, lipid-based formulations of amphotericin B show a broad spectrum of activity against pathogenic fungi, with a significantly higher success rate and a lower incidence of nephrotoxicity. However, an important disadvantage associated with lipid formulations is their increased cost [22].

Flucytosine (5-fluorocytosine), a halogenated pyrimidine, was reported as an antifungal in 1961 [23] and was later described as a promising agent against systemic mycoses due to *Cryptococcus neoformans* and *Candida albicans*. However, today its use has been restricted as an adjunct medication for the treatment of cryptococcosis in certain countries [24].

The discovery of the azole antifungal drugs, compounds with the generic suffix “conazoles”, was a significant accomplishment in the history of antifungal drug development [25]. Ketoconazole, a miconazole derivative, was introduced in 1981 and represents an advance in the search for new safe and effective agents for the treatment of systemic fungal infections as an oral agent. In a screening program, more than 100 triazoles derived from 2,4-dichlorophenyl were tested in mouse models of dermatophytosis and vaginal candidiasis, but only a few drugs entered the market [25].

Fluconazole, a triazole first described in 1985 by Richardson *et al*. as the main representative of azoles, became the most prescribed antifungal just a few years after being first used clinically; this drug is prescribed for almost all intensive care unit (ICU) patients with antifungal infections [26,27]. In that same decade, itraconazole was shown to be effective against *Aspergillus* spp. and agents of systemic mycosis such as paracoccidioidomycosis. This drug also has a high affinity for keratinous tissues, so it has been indicated for the treatment of onychomycosis and dermatophytosis [6].

A substantial increase in the number of antifungal drugs occurred at the beginning of this century with the availability of new azoles and echinocandins. Voriconazole was the main primary azole compound introduced as a drug and was approved for first-line treatment of invasive aspergillosis and also showed activity against *Fusarium* and *Scedosporium* infections, which are difficult to treat. Others new antifungals included triazoles, ravuconazoles and posaconazoles, although their discovery has not resulted in significant therapeutic gains [6,22]. Structurally, voriconazole and ravuconazole are related to fluconazole, whereas posaconazole closely resembles itraconazole [28].

In the early 2000s, the following three echinocandins entered the market: caspofungin, anidulafungin and micafungin. These drugs, which are natural products, are safe and well tolerated, and have minimal drug interactions and favourable pharmacokinetics [29,30,31]. Current guidelines recommend their use as first-line therapy for candidaemia/invasive candidiasis and as second-line drugs for aspergillosis because of their high activity against fluconazole-resistant *Candida* spp. and filamentous fungi [32].

Sordarins are a class of semi-synthetic natural products that are potent and selective inhibitors of fungal protein synthesis. A number of molecules based on the sordarin pharmacophore have demonstrated therapeutic efficacy in various animal models of fungal disease but are not yet available for clinical use [33,34,35,36,37,38].

Another promising new drug is E1210, which is an antifungal with a novel mechanism of action, the inhibition of fungal glycosylphosphatidylinositol biosynthesis. It has suitable *in vitro* activity against *Candida* spp. (including fluconazole-resistant strains), *C.* *neoformans*, and *Aspergillus* spp. and a wide range of medically relevant yeast and moulds [39,40,41,42].

Others promising drugs have not been used, and others were used but then fell into disuse due to toxicity, cost, spectrum of action, or unfavourable pharmacokinetics [43]. For every 5,000 to 10,000 experimental compounds tested, it is estimated that only one will obtain Food and Drug Administration (FDA) approval, which usually occurs after 10 to 15 years of research and development [44]. Therefore, extensive research is necessary and essential for the development of new antifungals that could support the combat antifungal resistance and have lower toxicity than current therapies. In addition, the development of new antifungals will allow elucidation of the underlying mechanisms and activity of the drugs to improve their use in medical mycology.

## 3. Natural Derivatives with Antifungal Properties

In addition to the development of synthetic antifungals, alternatives based on natural compounds, such as phenolic compounds, essential oils, and extracts from natural products, have been proposed. Furthermore, recent advances in structural biology, medicinal chemistry, and *in silico* technologies have allowed the development of promising research, including proposed antifungals based on targets unique to fungi to increase the specificity and reduce side effects [11,45,46,47,48,49,50].

When determining whether a natural product is an antifungal agent, it is important to consider the following microbiological parameters: (1) the use of a solvent without antifungal activity for compounds with poor solubility; (2) the best method selection, such as microdilution or macrodilution or broth or agar diffusion, according to Clinical and Laboratory Standards Institute Standards (CLSI) document M27-A3, M38-A and M2-A8; (3) careful observation of the rules for a standardised inoculum; (4) the use of reference strains according to the CLSI and pathogenic human fungi; (5) the use of a synthetic drug as the control; and (6) the appropriate incubation time. 

Thus, the next section will discuss preliminary research showing promising results for natural products with the potential to be novel antifungal drugs.

### 3.1. Extracts

Plant extract is a generic term for natural product derivatives with chemical components that have not been completely elucidated. Before performing research on the purification of extract components, it is important to screen the extracts for antifungal activity. 

Many types of extracts have been reported to show antifungal activity in the literature. Extracts derived from *Curcuma zedoaria* [51], *Psidium guajava* [52], *Plectranthus amboinicus, Aristolochia cymbifera*, *Plectranthus barbatus*, *Lippia alba*, *Hydrocotyle bonariensis*, *Hydrocotyle bonariensis*, *Justicia pectoralis* var. *stenophylla*, *Herreria salsaparilha*, *Mentha X piperita*, *Eleutherine bulbosa*, *Baccharis trimera*, *Calamintha adscendens*, *Albizia inundata*, *Bauhinia forficata*, *Cymbopogon citratus*, *Plectranthus grandis* [53] and *Euphorbia hirta* L. [54] have all demonstrated activity against *Candida* spp. More specifically, a study conducted by our group evaluated the antifungal activity of a hydroalcoholic extract of *Cymbopogon nardus* [49] in comparison with nystatin and fluconazole, and its impact on *Candida* virulence factors was evaluated. This extract was effective against *Candida* based on significant changes in the virulence factors, indicating a possible mechanism of action. In addition, a similar study conducted by Polaquini *et al*. [55] on a crude extract of Neem (*Azadirachta indica*), and the *Candida* virulence factors showed a decrease in yeast adhesion capacity, although this extract was not efficient in inhibiting the growth of *Candida* biofilm.

Another study of 46 *Eurasian area* derivatives and extracts from 25 plants belonging to the *Asteraceae*, *Euphorbiaceae*, *Rubiaceae* and *Solanaceae* botanical families [56] found good activity against filamentous fungi [57]. *Crossandra infundibuliformis* [58] and *Labisia pumila* Benth [59] are examples of extracts that showed both *Candida* spp. and filamentous antifungal activity. Extracts of Tanzanian-derived plants [60], *Alpinia galanga* and *Boesenbergia pandurata* were tested for activity against *Cryptococcus,* a fungi that is difficult to treat [61]. The extracts showed positive results, and these extracts also demonstrated activity against other fungi tested.

The biological activity of an extract may not be experimentally reproducible because the chemical constituents present in the crude extracts may be different. Furthermore, the solvent used to obtain the extract and the extraction method may result in the loss of some compounds. Some studies have shown changes in the chemical composition during different growth stages of plants, and others have shown diversity based on the geographic area of origin [62,63,64]. Although screening plant extracts can streamline the discovery process, studies have been focused on the elucidation of chemical compounds. Research on crude extracts may be the first step to discovering a new promising compound, which is followed by identification of the chemical compounds responsible for the antifungal activity.

### 3.2. Essential Oils

Essential oils are mixtures of volatile elements contained in diverse plant parts (flowers, leaves, bark, fruits and rhizomes). In most cases, these oils are obtained by steam distillation or by pressing the pericarp of citrus fruits. Essential oils are mainly composed of mono- and sesquiterpenes and phenylpropanoids (Section 3.2.1 Terpenes and Terpenoids), which confer its organoleptic characteristics, are connected with the various functions necessary for plant survival and play a key role in the defence of the plant against microorganisms [65,66,67,68].

The antimicrobial action of the different types of essential oils against microorganisms has been studied since the 1950s [69,70,71,72,73]. Viollon and Chaumont [74], using *C. neoformans* from an HIV-positive patient’s blood, evaluated the activity of some essentials oils and the pure constituents derived from them. Essentials oils from cinnamon, geranium “Bourbon”, geranium “Palmarosa”, savoury, and thyme showed good fungistatic action, but in general, the pure components (terpenoids, citral, geraniol, and citronellol) showed better activity than the essential oils.

*Ocimum gratissimum* is popularly used to treat many diseases. Eugenol is the main constituent of this essential oil and is considered to be responsible for its antimicrobial activity [75,76]. According to the *in vitro* minimum inhibitory concentration (MIC) values and time-kill curves demonstrated by Nakamura *et al*. [11], the *Ocimum gratissimum* essential oil had fungicidal activity against all *Candida* species; *C. parapsilosis* was the most susceptible and *C. tropicalis* was the least.

Other essential oils have been investigated. De Oliveira Lima *et al*. [77] evaluated the antifungal activity of *Cinnamomum zeylanicum* Blume, *Citrus limon* Risso, *Eucalyptus citriodora* HK., *Eugenia uniflora* L., *Peumus boldus* Benth. and *Rosmarinus officinalis* L. against *Candida*. They observed that all of these essential oils were effective in inhibiting at least one tested fungal strain. More specifically, *C. zeylanicum* and *P. boldus* showed the best results; they inhibited the growth of 58% of the tested strains. *E. uniflora* also showed low level inhibition against *Candida* strains.

Essential oils obtained from *Achillea millefolium* and *Curcuma longa* demonstrated substantial inhibition of the tested samples (*C. albicans*, *C. glabrata*, and *C. tropicalis*), at 63.2% and 68.4%, respectively [78]. The essential oil of *Nandina domestica* Thunb., which contains 1-indolizinecarbazole, 2-pentanone, monophenol, aziridine, methylcarbinol, ethanone, furfural, 1-hydroxy-4-methylbenzene, 2(5*H*)-furanone, and 3,5-dimethylpyrazole as major components (Table 1), exhibited high antifungal activity *in vitro* and had a strong detrimental effect on spore germination against *Microsporum canis*, *Trichophyton rubrum* and *T. mentagrophytes* [79].

Recent studies continue to demonstrate the antifungal activity of essential oils [80,81,82,83] and consequently increase the possibility of applying these products to the prevention and/or treatment of fungal infectious diseases. The biological activity of essential oils has been shown to be dependent on their chemical compositions, which include citral, pinene, cineole, caryophyllene, elemene, furanodiene, limonene eugenol, eucalyptol and carvacrol. These constituents are known to have antifungal activity, among other properties [84].

There are some clinical studies of essential oils. One example is the research conducted by Carmo *et al*. [81], which tested the essential oil of *Cymbopogon citratus* in shampoo and cream formulations to treat pityriasis versicolor. They proved that the formulations, which did not cause significant adverse reactions in healthy volunteers, were safe, and after treatment, a 60% mycological cure rate was observed for the group treated with this essential oil.

**Table 1:** Some essential oils, their main constituents and the fungus tested.

Essential oil	Main constituents	Fungus tested	Reference
*Calea clematidea*	Clemateol	*Trichophyton* *rubrum, T. tonsurans, T. mentagrophytes, Microsporum gypseum, M. canis, M. nanum, Epidermophyton floccosum*	[85]
*Ocimum gratissimum*	Eugenol	*C. albicans, C. krusei, C. parapsilosis, C. tropicalis*	[11]
*Salvia officinalis*	Camphor and *cis*-thujone	*C. albicans, C. krusei, C. tropicalis, C. parapsilosis, C. glabrata, E. floccosum, T. rubrum, T. mentagrophytes, M. canis, M. gypseum, A. flavus, Fusarium moniliforme, Penicillium italicum, Cladosporium cladosporioides.*	[86]
*Calendula officinalis* L.	δ-Cadinene, α-cadinol and *epi*-α-muurolol	*C. albicans, C. dubliniensis, C. parapsilosis, C. glabrata, C. tropicalis, C. guilliermondii, C. krusei, Rhodotorulla* sp.	[87]
*Nandina domestica* Thunb.	1-Indolizinocarbazole, 2-pentanone, monophenol, azridine, methylcarbinol, ethanone, furfural, 1-hydroxy-4-methylbenzene, 2(5*H*)-furanone, and 3,5-dimethyl-pyrazole	*T. rubrum, T. mentagrophytes, M. canis*	[79]
*Piper ovatum* Vahl	δ-Amorphene, *cis*-muurola-4(14),5-diene and γ-muurolene	*C. tropicalis*	[88]
*Piper amalago*	β-Copaen-4-α-ol, 7-*epi*-α-eudesmol, *epi*-α-cadinol and *n*-hexyl benzoate	*C. albicans, C. tropicalis, C. parapsilosis*	[89]
*Achillea millefolium*	Azulene, chamazulene, pinene, cineole, camphor, α-bisabolol, monoterpenes, eucalyptol, borneol, terpene derivatives, sesquiterpenes, tannins, mucilage, coumarins, resins, saponins, steroids, fatty acids, alkaloids, bitter principle, lactones and flavonoids.	*C. albicans, C. tropicalis, C. glabrata.*	[78]
*Curcuma longa* L.	Curcumin	*C. albicans, C. tropicalis, C. glabrata.*	[78]
*Boesenbergia pandurata*	Geraniol and camphor	*C. albicans*	[90]
*Coriandrum sativum* L.	Linalool	*C. albicans, C. tropicalis*	[91]
*Piper diospyrifolium*	(*E*)-Eudesma-6,11-diene, *γ*-muuroleneandcaryophyllene-type (*E*)-caryophyllene	*C. albicans, C. tropicalis, C. parapsilosis*	[92]
*Mentha piperita*	Menthol, menthyl acetate and menthofuran	*C. albicans, C. tropicalis, C. krusei, C. glabrata, C. dubliniensis, C. parapsilosis, C. neoformans, Aspergillus flavus, A. fumigatus, A. clavatus,* and *A. oryzae*	[93]
*Cymbopogon citratus*	Citral	*Malassezia* spp*.*	[81]

#### 3.2.1. Terpenes or Terpenoids

Terpenoids are some of the substances in essential oils [94], and terpene compounds from natural vegetable oils are frequently monoterpenes (approximately 90% of the volatile oil) and sesquiterpenes. Other terpenoids, such as diterpenes, are found only in volatile oils and can be extracted using solvents [94].

Studies have indicated that terpenoids may show antioxidant and antimicrobial properties against pathogenic fungi, including *Candida* and dermatophytes [95,96,97,98,99]. However, there are few terpene compounds with well-established antifungal activity and well-described pharmacological properties [94]. Machumi [100] studied the antifungal activity of nine diterpenoids from *Clerodendrum eriophyllum* against *Candida* spp*.*, *C. neoformans* and *A.* *fumigatus* and reported the isolation and structural determination of a new abietane diterpenoid (2-hydroxy-8,12-abietadiene-3,11,14-trione). They concluded that taxodione and 6-hydroxysalvinolone exhibited strong antifungal activity against *C. neoformans.* Furthermore, Amber *et al*. [101] observed that terpenes from *Ocimum sanctum*, including methyl chavicol and linalool, affected the synthesis of ergosterol and caused membrane cell damage in *Candida* species, which suggests that it may be a good antifungal agent. Isolated terpenes should be more thoroughly researched to establish the mechanism of their antifungal activity.

### 3.3. Propolis

Propolis is the generic name for a complex mixture of resinous substances collected from plants by bees. It is used in the beehive to coat the inner walls, to protect the entrance against intruders and to prevent the growth of fungi and bacteria [102]. The chemical composition of propolis is strongly influenced by the type of bee and vegetation and the season of the year [103,104]. Despite differences in propolis composition, studies at different times and in different regions have demonstrated its antifungal activity [105,106,107].

**Figure 1:**
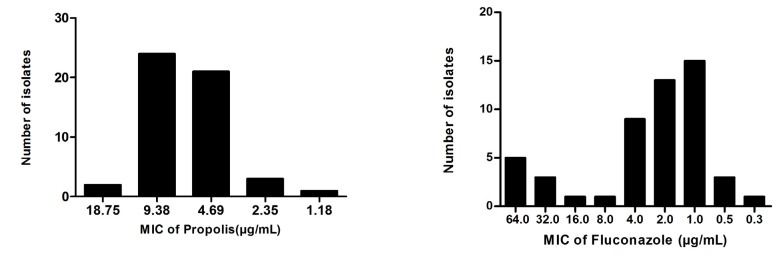
Results obtained by our group for propolis and fluconazole against *Candida* species isolated from HIV-positive patients.

Research has demonstrated the antifungal activity of propolis against fungi such as *Candida* spp. [105,106,108,109]. A study by our group that compared the activities of propolis and fluconazole against *Candida* spp. isolated from the mouths of HIV-positive patients showed that propolis extract was able to inhibit yeast with a MIC lower than that of fluconazole (unpublished results) (Figure 1).

Propolis also showed antifungal activity against dermatophytes [105,110], *C. neoformans* [111], and onychomycosis [110] and exhibited a synergistic effect with macrophages against *Paracoccidioides* *brasiliensis* [112]. Further, propolis has advantages such as a low cost and lack of toxicity [110].

However, for some preparations, such as ethanolic propolis extracts show certain disadvantages, as a strong and unpleasant taste, aromatic odour and high ethanol concentration. To reduce these disadvantages, Avanço *et al*. [113] developed a method to prepare propolis via a microencapsulation process and concluded that ethylcellulose microparticles containing propolis are useful for developing aqueous dosage forms of propolis that lack the strong and unpleasant taste, aromatic odour and high ethanol concentration from the ethanolic extract. Dota *et al*. [114] evaluated the antifungal activity after propolis microencapsulation and observed that propolis microparticles and the ethanol extract were able to inhibit the growth of yeast, including that of yeasts resistant to azoles.

### 3.4. Ajoene Derived from Garlic

The genus *Allium* contains over 300 plant species, and most of them belong to the Liliaceae family, which includes species of economic importance such as garlic (*A. sativum* L.), leek (*A. ampeloprasum* L. var. *Porrum* (L.) J. Gay), spring onion (*A. fistulosum* L.) and others. Currently, over 100 biologically active compounds have been derived from garlic, and most of them are organosulphur compounds, mainly ajoene. These compounds show a wide spectrum of activity that includes antimicrobial effects and beneficial effects on the cardiovascular and immune systems [115].

The major sulphur-containing compounds in garlic are *S*-allyl cysteine sulphoxide (alliin). When raw garlic is damaged, alliinase is released from the tissue and converted into primarily diallyl thiosulphinate (allicin) with a small amount of allyl methyl thiosulphinates. Ajoene [(*E*,*Z*)-4,5,9-trithiadodeca-1,6,11-triene-9-oxide)] is derived from allicin. However, efficiently obtaining ajoene is dependent on the specific processing conditions for the garlic, which also applies to the isolation of other bioactive compounds [116,117].

Ajoene shows important antifungal activity against both yeasts and filamentous fungi [118,119,120,121,122,123]. According to the literature, the filamentous fungi *Fusarium* spp. are 100% resistant to itraconazole [124,125,126]. Our group evaluated the *in vitro* effect of ajoene against *Fusarium* spp. and obtained satisfactory results (Table 2).

**Table 2:** Evaluation of the *in vitro* antifungal susceptibility of 27 onychomycosis clinical isolates of *Fusarium* sp. to ajoene compared to itraconazole.

Species (No. of Isolates)	MIC Range (µg/mL)	
	Ajoene	Itraconazole
*Fusarium oxysporum* (8)	14.63–29.25	16–16
*Fusarium solani* (8)	3.65–29.25	16–16
*Fusarium sacchari* (3)	14.63–29.25	16–16
*Fusarium verticillioides* (2)	14.63	16–16
*Fusarium chlamydosporum* (1)	29.25	16
*Fusarium proliferatum*(1)	14.63	16
*Fusarium incarnatum*(1)	29.25	16
*Fusarium napiforme* (1)	14.63	16
*Fusarium subglutinans* (1)	7.3	16
*C. parapsilosis* ATCC 22019 (1)	29.25	0.125

Notes: Concentrations from 0.03 to 16 µg/mL for itraconazole and 3.65 to 29.2 µg/mL for ajoene were used. The breakpoints used for itraconazole were ≤0.125 µg/mL, Susceptible strains; 0.25 µg/mL to 0.5 µg/mL, Susceptible dose-dependent strains; and ≥1.0 µg/mL, resistant strains. The reading was performed using a visual method, and the MIC was considered to be the concentration of ajoene or itraconazole that inhibited 100% of the growth of each yeast. Unpublished data obtained by Shinobu-Mesquita.

Other effects of ajoene were observed when tested against *P. brasiliensis*. It had the ability to block the transition from the mycelial phase to the yeast-like phase (an important mechanism in the pathogenicity of paracoccidioidomycosis) and efficiently controlled this disease in mouse experiments [127,128]. Furthermore, studies have shown that ajoene is effective for the topical treatment of superficial mycoses [129,130,131] and subcutaneous disease [132]. Thus, ajoene extract is an alternative for the treatment of the tested fungus and especially for onychomycosis caused by *Fusarium* spp.

### 3.5. Saponins

The name “saponin” is derived from *sapo*, the Latin word for soap, because these compounds have surfactant properties and form stable, soap-like foams when shaken in aqueous solution. These molecules are amphipathic glycosides with triterpene or steroid backbones. They are a large and structurally diverse group of bioactive natural products that are found primarily in plants, most commonly in dicots [133,134,135,136,137].

Many plants used in traditional medicine contain saponins, which are often responsible for the therapeutic action and protect against potential pathogens through their antimicrobial activity [138]. Saponins are extremely toxic to cold-blooded animals, but their oral toxicity in mammals is low [139,140]. Due to their toxicity to various organisms, saponins can be utilised for their insecticidal, antibiotic, fungicidal, and pharmacological properties. The wide chemical diversity of both triterpenoid and steroidal saponins has resulted in the renewed interest in and investigations of these compounds in recent years, particularly as potential chemotherapeutic agents [141].

**Figure 2:**
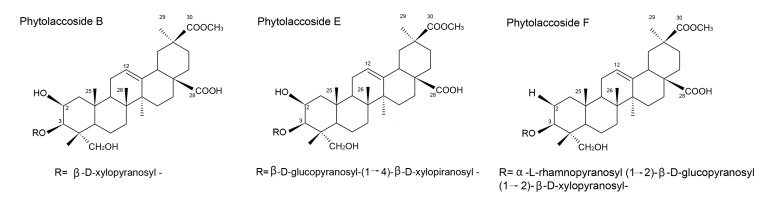
Isolated saponins of butanolic extract from Phytolacca tetramera. The triterpenoid saponins with one, two or three sugars as the glycon moiety were established as phytolaccosides B, E and F, respectively. Adapted from Escalante *et al*. [142], for illustrative purposes only.

The fractionation of the butanolic extract of the berries of *Phytolacca tetramera* Hauman yielded three monodesmosidic triterpenoid saponins with one, two or three sugars as the glycan moiety. The structures were established as phytolaccosides B, E and F (Figure 2). Phytolaccosides B and E showed antifungal activity against opportunistic human pathogenic fungi, and phytolaccoside B was the most active, mainly against *T. mentagrophytes*; in addition, it showed the broadest spectrum of action [142].

In 2010, Liberto *et al.* [143] studied the saponin-rich extract from *Phytolacca dioica* and its acid hydrolysate and major aglycone, as well as phytolaccagenin, and assayed their antifungal activity against *C. albicans* and *C. neoformans*. The extract showed no activity at concentrations up to 250 µg/mL, but the hydrolysate, which contained sapogenins and included phytolaccagenin, and pure phytolaccagenin showed promising antifungal activity.

The effect of the extracts of the aerial parts and roots of four *Medicago* species and 19 saponin compounds were studied by Houghton *et al.* [144]. All of the saponin extracts showed inhibitory effects on the growth of three dermatophytic species (*M. gypseum, Trichophyton interdigitale* and *T. tonsurans*), and *T. tonsurans* was the most susceptible*.* However, the aglycones showed less antifungal effect than the glycosides, which displayed a range of activity. The monodesmosidic glycosides of medicagenic acid were the most active compounds, particularly the 3-*O*-β-l-glucopyranoside, which displayed a MIC less than 0.09 mM against all three fungi. Hederagenin and zanhic acid also showed low levels of activity.

**Figure 3:**
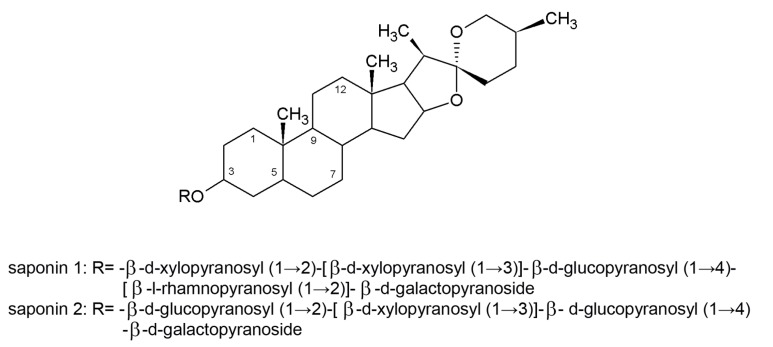
Two steroid saponins from *Tribulus terrestris* L. that show *in vitro* antifungal activity. These saponins are tigogenin-3-*O*-β-d-xylopyranosyl-(1→2)-[β-d-xylopyranosyl(1→3)]-β-d-glucopyranosyl (1→4)-[β-l-rhamnopyranosyl-(1→2)]-β-d-galactopyranoside (saponin 1), tigogenin-3-*O*-β-d-glucopyranosyl-(1→2)-[β-d-xylopyranosyl-(1→3)]-β-d-glucopyranosyl-(1→4)-β-d-galactopyranoside (saponin 2). Adapted from Zhang *et al*., [146], for illustrative purposes only.

Eight steroid saponins were isolated from *Tribulus terrestris* L., and their *in vivo* antifungal activities were tested against six fluconazole-resistant yeasts (*C. albicans*, *C. glabrata*, *C. parapsilosis*, *C. tropicalis*, *C. krusei*, and *C. neoformans*) [145,146]. Two compounds (Figure 3) were very effective against several pathogenic *Candida* species and *C. neoformans* [145]. Using phase contrast microscopy, Zhang *et al.* [146], demonstrated that saponin 1 (Figure 3) inhibited hyphal formation and destroyed the cell membrane. Thus, this saponin is therapeutically promising, particularly for patients who do not respond to conventional therapy.

Using the *Caenorhabditis elegans* (nematode) model as a heterologous host, potential antifungal natural products including the identified saponins were tested. The results demonstrated that certain saponins affected nematode survival comparably to amphotericin B, and two saponins (A16 (aginoside) and A19) inhibited *C. albicans* at low concentrations. Moreover, when used in combination with photosensitising compounds, the fungus displayed increased susceptibility to photodynamic inactivation due to the ability of the saponins to increase cell permeability, which facilitated the penetration of the photosensitisers [147].

The extract of *Sapindus saponaria* L. is rich in saponins and is being studied by a group of researchers at the State University of Maringá. The main components of the BuOH extract (BUTE) of the pericarp of *S. saponaria* (two acylated triterpene saponins, S1 and S2, and also an acyclic oligoglycoside) have been identified. The hydroalcoholic extract (HE) and BUTE demonstrated excellent inhibitory and fungicidal activity *in vitro* against *Candida* spp. [148]. Furthermore, Damke *et al.* [149] demonstrated that these extracts had strong *in vivo* antifungal activity against *Candida* species. To summarise, research on plants containing saponins is promising and suggests that they could act as new antifungal therapies.

### 3.6. Alkaloids

Alkaloids from plants are a diverse group and show a broad spectrum of activity. A basic nitrogen is the only unifying structural feature of this wide range of compounds. Typical alkaloids are derived from plant sources, contain one or more nitrogen atoms (generally in a heterocyclic ring), and usually show significant pharmacological activity [94,150].

The alkaloid 2-hydroxy-9-methoxyaporphine, isolated from the extract of *Beilschmiedia* species, showed potent activity against *C. albicans* with a MIC of 8 µg/mL [151]. Another plant, *Chelidonium majus*, contained the alkaloids 8-hydroxydihydrosanguinarine and 8-hydroxydihydrochelerythrine in its ethanol extract, and these compounds showed strong antifungal activity when tested against *Candida* spp*.* clinical isolates [152]. These alkaloids have MIC values that indicate antifungal activity, although more specific tests should be performed to confirm their safety and efficacy *in vivo*.

### 3.7. Phenolic Compounds and Flavonoids

Phenolic compounds are found in a large variety of plants, and they can be simple or complex structures containing at least one aromatic ring in which one or more hydrogens are substituted by a hydroxyl group. When the phenolic compounds are secondary metabolites of the plant, they are found to have a variety of structures, such as phenolic acids and coumarin derivatives and may be the water-soluble pigments of flowers, fruits and leaves. In addition, this class of compounds includes lignins and tannins, polymers with important functions in plants [94].

Phenolic compounds may be isolated from the ethanol extracts of different natural sources, such as plants, lichens and macroscopic fungi. Research conducted with *Cetraria pinastri* and *Parmelia crinita,* which are symbiotic organisms consisting of one fungi partner and one alga or Cyanobacteria (lichen) partner, showed a high level of phenolic compounds and activity against *C.* *albicans* and *Penicillium verrucosum* [153]. Among the compounds isolated from *Phellinus* spp., a macroscopic fungi, phenolic compounds showed substantial antimicrobial activity and inhibited the growth of *Alternaria alternata* [154].

Phenolic compounds from the extracts and essential oil of *Elais guineensis* showed antifungal activity against *C. albicans* when subjected to specific tests, including *in vivo* antifungal activity [155]. In another study, Kumar *et al*. [156] observed antimicrobial activity against *C. albicans*, *A. niger*, *A. flavus* and *Alternaria solani* for a methanolic extract from *Bauhinia racemosa* Lam, which contained 64.7 μg of pyrocatechol equivalents of phenols in 1 mg of extract. Moreover, the phenolic compounds eugenol, eugenyl acetate, β-caryophyllene, and 2-heptanone were isolated and characterised from the essential oil of *Eugenia caryophyllata* and exhibited antifungal activity against a large number of human pathogenic fungi [157].

Flavonoids are bioactive compounds with antioxidant and anti-inflammatory properties that are found in large quantities in certain plants as a result of their prevalence among secondary metabolites. These compounds represent one of the most important phenolic groups (polyphenols) and have a role in various functions, including the protection of plants against ultraviolet and visible light; protection against insects, fungi, viruses, and bacteria; the attraction of animals for pollination; and the action of plant hormones. In addition, they function as antioxidants, allelopathic agents and enzyme inhibitors [94].

Five prenylated flavonoids were isolated from the ethanol extract of the leaves of *Maclura tinctoria* (L.) Gaud and were evaluated against the AIDS-related opportunistic fungal pathogens *C. albicans* and *C. neoformans*. Two of these compounds showed inhibitory activity against *C. albicans* and *C. neoformans*, and another two were inactive against both organisms [158].

Another flavonoid, a flavone glycoside isolated from the ethanolic extract of *Vitex negundo*, showed significant antifungal activity against *T. mentagrophytes* and *C. neoformans* [159]. Curcumin, a flavonoid found in the *Curcuma longa* extract, showed antifungal activity against the yeast form of *P. brasiliensis.* In addition, this flavonoid was more efficient than fluconazole in inhibiting the adhesion of *Candida* species to human buccal epithelial cells, and it was able to inhibit the growth of *C. neoformans*, *Sporothirx schenckii* and clinical isolates of *C. dubliniensis* [160].

Finally, extracts from *Dorstenia mannii* (Moraceae), a medicinal herb traditionally used for the treatment of many diseases, contains the flavonoids dorsmanin A, B and C, 6,8-diprenyleriodictyol, dorsmanin E, F, G, and dorsmanin I, which were isolated and tested against yeast. All of these flavonoids and the crude methanol extract showed activity against *C. albicans* [161].

### 3.8. Coumarins and Xanthones

Coumarins are widely distributed in plants but can also be found in fungi and bacteria. Structurally, acid lactones are *O*-hydroxycinnamic acids, and the simplest representative is coumarin. Approximately 1,300 coumarins have been isolated from natural sources, and their pharmacological, biochemical and therapeutic applications are dependent on the particular substitution patterns [94].

Studies of three preparations from the essential oil of *Citrus bergamia* (bergamot natural essence, furocoumarin-free extract and the distilled extract) showed antifungal activity against dermatophytes and yeast pathogens [162,163,164]. Bergamot oil is directly obtained from the fruit and consists of a volatile fraction (93%–96%), whose main components are, with approximate percentages, limonene (40%), linalool (8%) and linalyl acetate (28%), and a non-volatile fraction (4%–7%) consisting primarily of coumarins and psoralens (*i.e.*, bergamottin, citroptene, bergaptene, *etc*.) [163,164]. The furocoumarin-free extract (bergaptene-free) and distilled extract (absolutely devoid of non-volatile residues) were more active than the natural essence against all of the species tested [162].

Xanthones are simple three-membered ring compounds that are mainly found as secondary metabolites in higher plants and microorganisms. Xanthones have very diverse biological profiles, including antihypertensive, antioxidative, antithrombotic, anticancer and antimicrobial activities, depending on their exact structures, which are diverse as a result of differences in the substituents on the ring system. The notable structural scaffold and pharmacological importance of xanthone derivatives have attracted many scientists to isolate or synthesise xanthone compounds for study as novel drug candidates [165,166,167].

Four xanthones have been isolated from the aerial parts of *Monnina obtusifolia*. The structures were established on the basis of their spectral data and that of their derivatives. One compound, 1,3,5-trihydroxy-2-methoxyxanthone, inhibited the growth of *T. mentagrophytes* and *Cladosporium cucumerinum*; another, 1,3-dihydroxy-2,5-dimethoxyxanthone, showed interesting activity against *T. mentagrophytes* [168]. Furthermore, some active xanthone derivative compounds showed good activity against fungi, making them promising candidates for further pharmacological and clinical studies [169,170].

### 3.9. Lignans

Lignans are phenolic plant compounds found in high concentrations in flax and sesame seeds and in lower concentrations in grains, other seeds, fruits and vegetables. Lignans are stereospecific dimers of cinnamic alcohols (monolignols) bonded at carbon 8 (C8-C8) that are derived in the phenylpropanoid pathway and formed through phenolic oxidative coupling processes. In the plant, lignans (monolignol dimers) usually occur in the free state or bound to sugars, and diglucosides of pinoresinol, secoisolariciresinol, and syringaresinol are common. Lignans, neolignans and their analogues are involved in plant defence (as antioxidants, biocides, phytoalexins, *etc*.), provide protection against diseases and pests and possibly participate in plant growth control [94,171].

Styraxjaponoside C is a glycoside derivative of lignans from the stem bark of *Styrax japonica* S. et Z and has shown activity against *C. albicans*, inhibiting mycelial growth with low cytotoxicity to human erythrocytes [172,173]. According to the literature, *Piper regnellii* roots contain several phenylpropanoids and dihydrobenzofuran neolignans, including (+)-conocarpan as the primary constituent. To investigate the antifungal activity of *Piper regnellii* leaf extracts, Koroishi *et al*. [174] evaluated the antidermatophytic activity of this extract as well as that of the isolated compound eupomatenoid-5 from the crude hydroalcoholic extract *in vitro* and observed that the pure compounds and eupomatenoid-5 showed strong activity against *T. rubrum*, indicating that the plant should be investigated for possible antifungal agents.

### 3.10. Tannins

Tannins are particularly important gustatory components that are responsible for the astringency of many fruits and vegetables. Tannin-protein binding is the basis for the ability of tannins to control insects, fungi and bacteria as well as their pharmacological activities [94].

Phytochemical investigation of *Terminalia mollis* and *Terminalia brachystemma* resulted in the isolation of many compounds, including the tannin ellagitannin punicalagin, which showed activity against *C. parapsilosis*, *C. krusei* and *C. albicans* [175].

*Stryphnodendron adstringens* (Mart.) Coville, Leguminosae occurs in the central region of Brazil. Stem bark from this plant is popularly used as an anti-inflammatory and astringent and in the treatment of wounds and vaginal infections [176,177]. The antifungal activities of a crude extract and the fractions and subfractions from *S. adstringens* (Mart.) Coville were investigated. These extracts showed moderate antifungal activity against *C. albicans* and low cytotoxicity to murine melanoma cells [178]. This extract was also assayed against *C. neoformans* [179] and interfered with important factors for virulence, including growth, capsule size, and pigmentation.

### 3.11. Secondary Metabolites

A large number of products derived from the secondary metabolism of various microorganisms have been reported, and many of them are being exploited by the pharmaceutical industry as potent antibiotics [180,181]. Thus, it is important to emphasise the findings of Phongpaichit *et al*. [182], who studied endophytic fungi from *Garcinia* spp. and observed that the secondary metabolites from this genus showed antifungal activity against relevant pathogenic fungi (*C. albicans*, *C. neoformans* and *M. gypseum*)*.* Another group studied the marine actinomycete *Nocardia dassonvillei* and identified a secondary metabolite, *N*-(2-hydroxyphenyl)-2-phenazinamine (NHP) [183], with significant antifungal activity against *C. albicans*.

Xu *et al*. [184] evaluated the chemical composition and bioactivity of the extract from *Paecilomyces* sp. isolated from *Panax ginseng* and its activity against *T. rubrum*, *C. albicans*, *C. neoformans* and *A. fumigatus*. This extract showed activity against two of these fungi, but it was not effective against *A. fumigatus*. Extracts derived from *Paecilomyces* sp. and ginseng samples contained the same compound, falcarinol, providing evidence that endophytes produce metabolites which are the same or similar to those of their hosts.

## 4. Perspectives

As previously discussed, many available drugs, including antifungals, were discovered from random observations and/or a search of synthetic or natural compounds [45]. Studies of structure-activity relationships permitted chemical modifications, generating new compounds with improved pharmacological characteristics. Historically, the drug-development process has utilised high-throughput screening (HTS), in which large number of compounds are evaluated to identify those that are biologically active [185,186].

Some studies utilised the HTS strategy to develop new antifungal agents. β-1,6-glucan synthesis inhibitors, including the pyridobenzimidazole derivative D75-4590, were obtained using high-throughput methodologies with a cell-based assay system and a chemical library of approximately 100,000 small molecules. These compounds were only active against *Candida* species [187]. In 2010, two compounds derived from D75-4590 were obtained, D11-2040 and D21-6076. Fungistatic activity was observed against *Candida* species, primarily non*-C. albicans* *Candida* (NCAC). However, no significant inhibitory effects were observed against *C. neoformans*, *Trichosporon* spp. or filamentous fungi [188]. Takeshita and colleagues identified another pyridobenzimidazole derivative with antifungal activity. An SAR study revealed that a basic substituent at the C-1 position and a cyano group at the C-4 position were essential for antifungal activity; however, these compounds were only active against NCAC species [46]. Kuroyanagi *et al.* [47] synthesised triazolopyridine derivatives with antifungal activity against NCAC species, especially *C. glabrata*. The development of β-1,6-glucan synthesis inhibitors has shown promise, particularly for species resistant to conventional antifungal agents in candidaemia treatment, although their use is restricted to *Candida* species.

Other benzimidazole derivatives have been identified by HTS methodologies. (*S*)-2-Aminoalkylbenzimidazole derivatives were found to be effective against *Candida* spp. at low micromolar concentrations using an HTS strategy with an activity-selectivity assay, which analyses both the antifungal activity and compatibility with HeLa cells in the same assay. Structure-activity relationship studies showed that the (*S*)-stereoisomers had effective antifungal activity [189]. Complementary studies of this group [190] showed that this new lead structure has high antifungal activity against clinical isolates of several *Candida* species. In addition, transcriptional profiling has shown that this compound is a potential inhibitor of the ergosterol pathway, in contrast to other benzimidazole derivatives, which target microtubules.

Chaturvedi *et al*. [191] selected 27 compounds with antifungal activity against *Geomyces destructans*, the etiologic agent of geomycosis in bats, from a library of 1920 structurally diverse compounds (drugs, experimentally active compounds, and pure natural products). Some molecules showed significant antifungal activity, but this information was not novel because the selected compounds had previously been described as antifungals, fungicides and biocides. Similarly, other cases have been reported in which HTS methodologies have been used for the rapid identification of potential therapeutic agents from libraries of known drugs and pharmaceutically active compounds that have been approved by the FDA [192].

Even with the evolution and emergence of new methodologies for HTS, these techniques are time consuming and expensive, especially when the tests are based on cellular assays [193]. Various computational approaches are available to contribute to HTS technologies. Among these, virtual screening (VS) is one of the most popular. This *in silico* methodology contributes to the research on and development of new drugs by allowing the efficient screening of millions of chemical compounds in libraries at reduced cost [194,195,196]. Additionally, VS technology allows the development of compounds that are different from those that have been patented. Thus, alternatives can be found without performing HTS and experimentally testing thousands of compounds [197].

In the post-genomic era, the advent of new technologies has generated an extraordinary amount of data and created more possibilities for drug development [45,198,199]. The identification of the molecular targets of antifungal drugs and elucidating their mechanisms of action in the fungus is an important undertaking not only for the development of novel drugs but also for the development of new pharmacological probes that can be used to investigate the basic biology of fungal pathogens [199,200]. After a new molecular target has been determined, new therapeutic agents may be identified by analysing the interaction of the candidate compounds with three-dimensional models of the target proteins [201,202].

Between 1999 and 2008, the FDA approved 183 small-molecule drugs. Despite the possible limitations of a target-centric approach to drug discovery, these approaches led to the discovery of 17 of the 50 first-in-class small molecule new molecular entities (NMEs). However, the approved small molecules do not include antifungals [203]. New antifungal development by this approach still seems to be in the preliminary stages, given that few studies are found in the literature, and those that exist are at the basic research stage [204,205,206].

Virtual screening studies have been used to select inhibitors of the CYP53 family of cytochrome P450 enzymes, which do not have a homologue in higher eukaryotes. From the 143,000 compounds selected for docking experiments, eight compounds showed promising results in *in vitro* assays. The best compound was (3-methyl-4-(1*H*-pyrrol-1-yl)benzoic acid, which was predicted to be an inhibitor of a fungal-specific enzyme and was thus a promising new lead for antifungal development [204]. However, these studies are still preliminary, considering that the antifungal activity assays were performed only with *Cochliobolus lunatus* using agar diffusion methods. Thus, additional experiments showing antifungal activity against several human pathogens by standardised methodology are still necessary. Sheng and collaborators identified four compounds that are more potent than fluconazole, itraconazole, and voriconazole against *C. albicans* and are active against other important human pathogenic fungi (*C. tropicalis*, *C. neoformans* and dermatophytes species). These results reveal the efficiency of the structure-based optimisation of novel azole antifungal agents and indicates that they are promising leads for the design of new drugs selected by VS strategies [207].

Recent technological advances have yielded increasing amounts of available data, not only of genomes and proteomes but also of host-pathogen interactions and the mechanisms of drug action. Therefore, rational antifungal drug design is possible. These new approaches have contributed during different stages of antifungal development because they optimise existing compounds to direct the process (new drug against new target). Researchers have primarily attempted to optimise the currently available drugs, especially the azole class, using molecular chemistry and bioinformatics [208,209,210]. Other investigators have focused on novel drug targets for the development of therapeutic options to obtain more specific compounds [211,212]. Additionally, advances in biotechnology and nanotechnology have contributed to the development of drugs that have already aided in the treatment of invasive fungal infections with worldwide relevance [213,214,215].

## 5. Conclusions

In summary, there are many on-going efforts in the search for new compounds with potential antifungal activity. Some of these compounds may become future prototypes of new antifungal drugs. However, more research is needed to ensure reliable results. There are diverse techniques for obtaining extracts from plants; these exhibit differences in the extraction parameters, including the solvent used, thus generating aqueous or hydroalcoholic mixtures, and utilise various extraction procedures, including infusion, decoction or maceration. These technical approaches have allowed compounds with different chemical characteristics to be isolated. Therefore, it is difficult to compare studies. Interlaboratory variability is also an issue.

Similarly, the reported antifungal actions seem to be satisfactory, but characteristics of the natural products influence the assays used to determine the antifungal activity. Because there are no standard protocols, researchers modify the procedures used in previous studies; however, each group focuses on what is important to that individual group; thus, the results obtained are not comparable. For example, we found publications using the agar diffusion test, which is ineffective because the molecules tested have different diffusion properties. Thus, it is impossible to evaluate the results of this assay and determine the antifungal activity.

It is interesting to note that the studies have generally been conducted on a few fungal isolates; therefore, it is necessary to perform further testing with a larger group of fungi including various genera and species of yeasts and filamentous fungus of medical interest. These complementary studies are essential because the antifungal action of a drug may vary according to the fungal species. This variation could result in treatment failure when the identification of the agent responsible for the infection is performed incorrectly and, consequently, incorrect drugs are used. Another problem is the selection of the species for the antifungal activity assays. If the focus is the treatment of human and/or animal mycoses, these assays should be carried out using the human or animal pathogenic fungi, at least for the most prevalent diseases. However, many studies have used saprophytes or environmental fungi in their antifungal activity evaluations.

It is important to highlight the importance of reproducibility and the correlation between the *in vitro* and *in vivo* activity. *In vivo* studies simulating fungal infection could confirm the antifungal action of these compounds, and studies of pharmacokinetics and pharmacodynamics could indicate the effectiveness of a possible treatment. There are few studies on conventional or alternative animals using natural products.

Another critical problem concerns the complexity of the natural products that have been evaluated. In general, they are complex mixtures from crude extracts or essential oils. It is important to isolate the specific compound responsible for the antifungal action and elucidate the mechanism of action of each isolated compound. It would be interesting to know if a specific compound acts on the cell wall or the cytoplasmic membrane or if it inhibits a specific pathway. New molecules open up new possibilities, and molecules capable of acting on specific targets that are not shared with the host would reduce the cytotoxicity and consequent side effects. This requires understanding the mechanism of action of the compound.

Current strategies for the development of new antifungal agents by biotechnology-related approaches seem promising, but these techniques are time consuming and expensive. They are also in their infancy for use in antifungal drug development, given that few studies are found in literature.

Finally, all of the studies cited have great potential for the development of new drugs, but most studies have been performed with molecules, extracts, and oils. Research regarding the toxicity of these compounds must be emphasised, especially concerning their pharmacodynamic characteristics and the potential implications of pharmacogenomics in predicting responses to these treatments. In other words, the development of new options for antifungal drugs is mandatory in view of the increase in fungal infections and their mortality rates. This process is a challenge to researchers and the pharmaceutical industry, but the recent application of biotechnological approaches has facilitated new discoveries.
